# The economic benefits of increasing kangaroo skin-to-skin care and breastfeeding in neonatal units: analysis of a pragmatic intervention in clinical practice

**DOI:** 10.1186/s13006-015-0035-8

**Published:** 2015-03-20

**Authors:** Karin Lowson, Clare Offer, Julie Watson, Bill McGuire, Mary J Renfrew

**Affiliations:** York Health Economics Consortium, Enterprise House, University of York, York, YO10 5NQ UK; Specialty Registrar in Public Health, University of York, Heslington, York, North Yorkshire YO10 5NH UK; Department of Nursing and Midwifery, Faculty of Health and Wellbeing, Sheffield Hallam University, Robert Winston Building (F414), Collegiate Crescent Campus, Sheffield, South Yorkshire S10 2BP UK; Hull York Medical School/NIHR Centre for Reviews and Dissemination, University of York, Heslington, York, North Yorkshire YO10 5NH UK; University of Dundee, School of Nursing & Midwifery, 11 Airlie Place, Dundee, DD1 4HJ UK

**Keywords:** Quality improvement, Change at scale, Breastfeeding, Kangaroo care, Skin-to-skin, Kangaroo skin-to-skin, Costs, Benefits, Economics, Neonatal, Preterm

## Abstract

**Background:**

A number of significant recent research studies have used techniques of economic modelling to demonstrate the potential benefits of increasing breastfeeding rates in the UK overall, and specifically in neonatal care. This paper complements this growing body of evidence by presenting an economic analysis of data from an actual intervention, the ‘Getting It Right From the Start’ programme, which took place in the north of the UK during 2011–12, with the aim of increasing breastfeeding and kangaroo skin-to-skin care rates in neonatal units.

**Methods:**

‘Getting It Right from the Start’ was a pragmatic, multifaceted programme of change delivered under the auspices of the regional Health Innovation and Education Cluster, of which 17 were established in the UK in 2010. It engaged with 18 neonatal units in two Neonatal Networks with the aim of increasing kangaroo skin-to-skin care and breastfeeding rates.

As part of the evaluation of the programme, we conducted an economic study comparing the overall costs and benefits of the intervention.

**Results:**

Overall, the economic analysis demonstrated that for every £1 invested in the intervention to increase kangaroo skin-to-skin care and breastfeeding rates, between £4.00 and £13.82 of benefit was generated. This was spread across different healthcare settings and the timescale for the realisation of benefits will vary.

The increases in kangaroo skin-to-skin care generated the greatest cost savings, with potential cost savings ranging between £668,000 (minimum cost assumptions) to more than £2 m (maximum cost assumptions).

Increases in breastfeeding associated with the project generated between £68,486 and £582,432. The majority of the cost savings generated were associated with reductions in cases of gastroenteritis and necrotising enterocolitis.

**Conclusion:**

This was one of the first economic evaluations of an actual intervention to increase breastfeeding and kangaroo skin-to-skin care in neonatal units. It complements the existing economic models by demonstrating that a real intervention in clinical practice was both cost effective as well as clinically beneficial. Future interventions with similar methodology should be supported and considered likely to generate significant cost savings compared to outlay.

Economic evaluation should be more frequently included in studies of practical interventions in clinical settings to increase breastfeeding.

## Background

Recent major studies from the UK, US, the Netherlands and Australia [1-5] have modelled very significant economic benefits for increases in breastfeeding rates overall, and for increases in breastfeeding in neonatal care. Kangaroo skin-to-skin care is recognised by the above research as a significant factor in promoting breastfeeding, and hence a contributor to its economic benefits. Kangaroo skin-to-skin care also has independent benefits, the economic impact of which has not yet been modelled.

This paper builds on previous research by presenting an economic evaluation of an actual intervention in clinical practice, comparing the costs and benefits of that intervention. In 2011 and 2012, the ‘Getting It Right from the Start’ programme was delivered in Yorkshire and the Humber, which aimed to increase rates of kangaroo skin-to-skin care and breastmilk feeding at discharge in 18 neonatal units across two Neonatal Networks. Kangaroo skin-to-skin care has significant independent benefits relating to attachment and psychological wellbeing, as well as being an important factor in successful breastfeeding [6,7]. The programme was successful in demonstrating significant changes in the numbers of babies receiving kangaroo skin-to-skin care, and more modest changes in breastfeeding rates at discharge. Hence it is possible, in this paper, to complement the understanding from economic models with an example of the potential savings accruing from an actual intervention.

### ‘Getting it right from the start’

The ‘Getting It Right From the Start’ programme (GIRFS) was a pragmatic, multifaceted programme of change delivered under the auspices of the regional Health, Education and Innovation Cluster (HIEC). HIECs were first mooted in the Darzi report [8], which described them as associations made up of:

‘… many partners, across primary, community and secondary care, universities and colleges, and industry … Their members will run joint innovation programmes that reflect their local needs and distinctiveness. They will also promote learning and education between their members. Bringing NHS organisations and higher education institutions together will enable research findings to be applied more readily to patient care.’ (p.56) [8].

Seventeen HIECs were eventually established in the UK with a variety of partners, but all aiming to bridge the gap between academic research findings and practical, front line patient care. The Yorkshire and Humber programme included three separate ‘strands’ under an overarching project board: Patient Safety, Long Term Conditions, and Maternal and Infant Health and Care (MIHC). The MIHC theme, with which this paper is concerned, was led by the Mother and Infant Research Unit at the University of York. Further description of the structure and processes of the Yorkshire and Humber HIEC is available in their published evaluation report [9].

The methodology adopted by the MIHC theme was grounded in earlier research by Renfrew et al. [10] and Dyson et al. [11]. A detailed description of the methodology underlying the programme of work is available in the published report of the theme’s initial consultation [12], the published final report of the work of the HIEC [13], and in forthcoming papers. A description of the programme in practice in one neonatal unit has recently been published [14].

The programme was fully supported by the two Neonatal Networks, the region’s strategic health authority, senior staff in all the participating National Health Service (NHS) trusts, and by the audit and clinical governance teams in each trust. Its initial aim was to identify and prioritise evidence based interventions which stood the best chance of increasing breastfeeding rates, and bonding and attachment in neonatal units in the region.

A systematic review was undertaken [15], to identify a range of evidence-based practices that would promote these aims. These interventions were then included in a web-based questionnaire that formed the basis of a consultation conducted with practitioners, managers, commissioners, and user/advocacy groups prior to the set-up of the programme [12]. Face to face workshops were also delivered [12].

Participants were asked to prioritise these interventions on the basis of whether they were ‘high impact’ (likely to significantly change care) and ‘high feasibility’ (relatively simple to implement) in neonatal units. Two key practices were identified as the focus of the programme; kangaroo skin-to-skin care, and early support for breastfeeding women, including (but not limited to) teaching techniques of hand expression and increasing mothers’ access to ‘double pumping’ with an electric pump.

The next step in the GIRFS programme was to engage with and support neonatal units in delivering those interventions identified as priorities. Implementing change ‘at scale’, rather than in selected pilot sites, was a key principle, meaning that all neonatal units were offered the opportunity to participate. The exact level and type of activity was highly dependent on the individual unit, as the paragraph below explains.

The second key principle of the programme was that it should not be implemented from the top down, but should be developed from within the units and tailored to their own needs. GIRFS was comparatively resource-light, relying on only three specialist staff who were also working across other areas of the programme. The staff allocation to the neonatal area of the programme was approximately 1.5 whole-time equivalents (WTE). Thus each unit that committed to taking part was asked to identify a champion (a senior staff member with responsibility for leading the HIEC MIHC work) and any number of enablers (staff at any grade who were committed to spreading the word and being advocates for best practice). The approach of the team was to develop these staff, put them in touch with one another, and give them the necessary tools to do the job themselves.

More than 120 staff members across 42 maternity and neonatal units were recruited and trained as HIEC MIHC ‘champions’ and ‘enablers’, the NHS partners providing the onsite training venues. Attendance at these workshops and development days was deemed part of staff members’ continuous development programme.

The programme began with a series of development days for champions and enablers, and clinical skills training days for professionals. The development days were intended to develop generic skills in leadership and change, so that staff could design and cascade the change programme in their own settings. The clinical skills training days were intended to provide staff with the detailed knowledge and skills needed to change practice in complex areas. The ‘Getting It Right from the Start’ programme involved 6 development days and 7 clinical skills days, with more than 200 attendees in total. Subsequently, the programme team offered individualised support to each unit in the region, including individual visits, email and telephone support.

Enabling participants to form links, discuss their experiences and share best practice was also a key aim of these days, and throughout the programme. An electronic network of communication was set up via the HIEC MIHC website, providing access to shared resources and learning, and offering an online forum for staff to exchange ideas. This network now has more than 1600 staff members signed up. A wide range of external collaborations and partnerships were fostered at a local, regional and national level including work with the charity ‘Best Beginnings’ on a pilot DVD [16], and work with the two neonatal networks in the region and the local Infant Feeding Co-ordinators network.

Detailed action plans were the responsibility of the individual units, with support from the GIRFS team, as described above, and included extra support and training for staff, the production of clinical guidelines, investment in additional equipment and awareness raising measures.

The impact of the GIRFS programme was evaluated by an independent team from the National Child and Maternal Health Intelligence Network (ChiMat), and the University of York’s Health Sciences Department and York Health Economics Consortium (YHEC). It is the economic elements of the evaluation that form the basis of this paper.

### Kangaroo skin-to-skin care

Skin to skin care, also known as ‘kangaroo care’ or ‘Kangaroo Mother Care’ was first described in Bogota, Colombia in 1978. It involves skin-to-skin positioning of the baby in an upright position on the mother’s chest. The baby can be cared for in this way continuously (for more than 20 hours/day) or intermittently (for periods of hours at a time) [6]. Although kangaroo care came to prominence in the developing world as a safe and effective alternative to scarce neonatal cots, it is also recognised as a helpful intervention in developed countries and high-tech neonatal environments [6].

For the purposes of this programme of work, we defined ‘kangaroo skin-to-skin care’ as a period of care where the infant is held upright between the mother’s breasts or on the father’s chest, undressed and receiving direct skin-to-skin contact. We measured any skin-to-skin contact which occurred for at least a ten-minute period, although in most cases periods of skin-to-skin care went on for much longer.

It has not been conclusively shown that kangaroo skin-to-skin care has an impact on infant mortality [7], but it has a positive effect on morbidity. Babies in receipt of intermittent and continuous kangaroo skin-to-skin care appear to suffer fewer severe infections or sepsis, severe illness at six months follow-up, nosocomial infections, lower respiratory tract disease at six months follow-up, and hypothermia at discharge [7]. Early kangaroo skin-to-skin care also has a positive effect on uptake and duration of breastfeeding [15,17].

The use of kangaroo skin-to-skin care has a beneficial impact on the management of babies. Infants receiving kangaroo skin-to-skin care are discharged home from hospital earlier and spend less time in hospital at one year gestational age [18]. The review by Conde-Agudelo and colleagues [7] found that kangaroo skin-to-skin care decreased length of hospital stay by 2.4 days in studies using intermittent kangaroo skin-to-skin care. In a study by Charpak et al. [13] the mean hospital stay at 41 weeks gestational age was 4.5 days for infants having experienced kangaroo skin-to-skin care compared to 5.6 days in control infants, whilst Cattaneo et al. [19] reported a median hospital stay of 11 days in the kangaroo skin-to-skin care infants compared to 13 days in the control.

Hall and Kirsten [18] reported that fewer infants with mild or no respiratory distress symptoms who received kangaroo skin-to-skin care and were fed breastmilk in a Level 2 neonatal unit received fewer packed red cell transfusions compared with a control group. Total transfusion costs decreased by 55% even though the number of patients increased by 19%. Where preterm babies have to be transported between units, movement using kangaroo skin-to-skin care appears to be better, with heart rate, respiratory rate and oxygen saturation of babies transported remaining stable during journeys of between 10 minutes and five hours [20].

A number of research studies have demonstrated, as have parental interviews from the GIRFS project, that kangaroo skin-to-skin care promotes bonding, improves parent-infant attachment, positively affects mother and baby relationships and helps new parents gain confidence in their role [21-23]. However, for the purposes of the economic analysis we have not placed a monetary value on these benefits.

### Breastfeeding

In our study, we aimed to increase the proportion of babies who were breastfed during their time on the neonatal unit and at discharge. We defined this as the proportion of babies receiving any breastmilk at discharge from the neonatal unit, including mixed feeding and the feeding of expressed breastmilk by means such as cup or bottle.

For preterm babies and babies who have growth restrictions or are sick, evidence indicates that the use of breast milk substitutes is associated with adverse short and long term outcomes, including mortality and serious morbidity, which can be costly for the NHS and for families [15]. The incidence of invasive infection is higher in low birth weight babies who are fed formula feeds. These babies are five times more likely to suffer necrotising enterocolitis (NEC), with one-fifth of babies who are affected by the condition dying, and those who survive being more likely to have longer term health problems [15].

Breastfed babies have greater protection against hospitalisation for diarrhoea and lower respiratory tract infection [24], and breastfeeding is protective against wheeze in the first three years of life. Breastfeeding also appears to have a role in the prevention of middle ear infection, urinary tract infection, juvenile onset insulin-dependent diabetes mellitus, raised blood pressure and asthma [25,26]. Breastfeeding also confers benefits on the mother: women who do not breastfeed are more likely to develop epithelial ovarian and breast cancer [27].

## Methods

The aim of the economic evaluation was to translate the outcomes and benefits from the programme as demonstrated in the data on outcomes, into economic benefits which could then be compared to the costs of the intervention. Detailed descriptions of the economic methods used to undertake the analysis are available in the full published evaluation report produced by the York Health Economics Consortium (YHEC) [28].

The underpinning body of evidence cited above included data and information that could be used for the economic analysis, such as quantification of expected outputs and outcomes, and changes in resource utilisation expressed as cost-savings. Published evidence on the impact of breastfeeding and kangaroo skin-to-skin care on morbidity in babies was used to model the likely reduction in health services utilisation as breastfeeding rates increased, to which costs were applied. The published evidence for the benefits expressed as cost savings are calculated using similar, but slightly different diseases, and therefore we have calculated and applied different cost-savings accordingly. We did not apply cost-savings directly to intangible, but important, outcomes such as family cohesion. The perspective of the study was from that of a health provider and only health service costs have been included.

The benefits as expressed as cost-savings were derived from the national reference costs calculated and published by the UK’s Department of Health using data collected from all NHS hospitals and organisations [29], unit costs of health and social care calculated and published by the Personal Social Services Research Unit of the University of Kent using data collected from all NHS hospitals and organisations [30] and from the published literature. Relevant uplifts were applied to costs to bring them up to a consistent price base of 2010–11. Costing methodologies used a range of costs where appropriate, for example, the minimum and maximum. Modelling of alternative options for changes in the impact of the intervention was also undertaken where relevant, and appear in the published economic evaluation [28].

We estimated the notional cost burden falling on the NHS partners as a result of around 200 staff members attending the workshops and training days to be £50,000. However, we have not included these costs in our cost benefit calculations as funds were available from HIEC to reimburse organisations, which were not taken up. The organisations treated all training as legitimate continuous professional development, and any teaching input from the neonatal network educational leads was also deemed to be part of their normal role. Copies of the *Small Wonders* DVD [16], produced by the Best Beginnings child health charity which informed the evidence based training resource, were distributed freely to all participating neonatal units from the start of the pilot project. Parents who attended any of the workshops (we do not have the numbers) were paid local travelling expenses, again from HIEC funds.

### Benefits and cost savings for infant outcomes – kangaroo skin-to-skin care

Data informing the economic study were collected as part of the overall evaluation of the MIHC theme. Because there were no routinely available data on kangaroo skin-to-skin care, the GIRFS programme instituted a local audit, in which participating neonatal units submitted data on a weekly basis. For a randomly selected 24 hours each week they returned information about the babies in their care who had received any skin-to-skin care. Around 4,000 babies were audited with an average of 125 babies involved in the audit each week.

**Figure 1:**
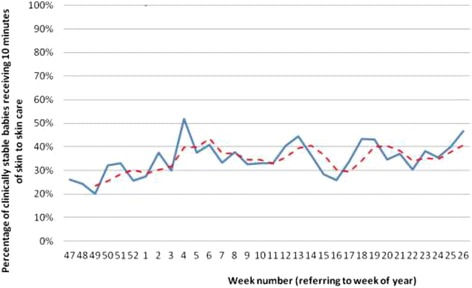
**The proportion of clinically stable babies receiving at least ten minutes of kangaroo skin-to-skin care, Yorkshire and the Humber, 2011/12.** The solid line represents actual figures each week while the dotted line shows a rolling average over 3 weeks.

Figure 1 shows the increase in babies receiving at least ten minutes of kangaroo skin-to-skin care during the GIRFS programme. Between the first and last weeks of the intervention it demonstrates a statistically significant overall increase of 20% in the proportion of babies receiving skin-to-skin care (95% CI 7%-32%). We estimated how many additional babies would receive kangaroo skin-to-skin care each year, if the proportional increase shown in Figure 1 were maintained across the region.

Data provided by ChiMat (the National Child and Maternal Health Intelligence Network, www.chimat.org.uk) [31] allowed us to estimate that approximately 8,000 babies a year were cared for in neonatal units across the Yorkshire and Humber region. At the pre-intervention baseline level, 26% of these babies would be likely to receive kangaroo skin-to-skin care, equating to 2,080 babies. If the increase achieved by GIRFS is sustained, we could expect that proportion to rise to 46%, equating to 3,680 babies each year. However, in order to allow for limitations of the local audit data, discussed further below, this figure was then reduced by 50% to arrive at a conservative estimate.

The economic analysis was conducted on the basis of 800 additional babies per year receiving kangaroo skin-to-skin care as a result of the MIHC intervention. Economic benefits accrued from the impact of the intervention on length of stay in neonatal units for babies without infections, on rates of morbidity, and on the need for and management of babies during transfer between units.

Evidence on the impact of kangaroo skin-to-skin care on length of stay in neonatal units indicates that length of stay can be reduced by between 1.1 days and 2.4 days [7,13]. We calculated the potential savings for a range of illnesses, for which there is clear evidence in the published literature: namely severe infections, general illnesses, general infections and respiratory infections, assuming a reduction in the number of episodes and length of stay in neonatal units [7,13], to which we applied the relevant inpatient cost as derived from the Department of Health’s reference costs. We have also made assumptions about the reduction in the number of babies requiring critical care transport, which were already low. We were aware of the potential for double counting; for example, evidence on reductions in length of stay at neonatal units may be in part a result of reductions in morbidity, the impact of which we have calculated separately. This is discussed in more detail in the economics report [28].

### Benefits and cost savings for infant outcomes – breastfeeding

To assess the impact of the programme on breastmilk feeding at discharge, we used routine data available through BadgerNet [32], the main clinical record keeping system for neonatal units in the UK. We observed rates to rise from 46% to 47% in one neonatal network, and from 40% to 52% in the other. However, in the network showing the most significant rise, almost 20% of records did not have feeding information completed prior to the GIRFS intervention. Post-intervention, the dataset was almost complete. This makes it difficult to be sure how much of the apparent increase may be attributable to the GIRFS programme, and how much to better ascertainment of mothers who were already breastfeeding. Because of the uncertainty around the data, a sensitivity analysis was conducted, using two further sets of assumptions based on an increase to only 45% or 50% for breastfeeding in the second network.

For the purposes of the economic analysis, we assumed that the entirety of the increase was attributable to the intervention, although we recognise that other factors are likely to have had an impact in the same timescale. To address this uncertainty, we have undertaken sensitivity analysis on the observed increase in the network with the significant rise to ensure that we have not overestimated the impact of the intervention.

We calculated the economic benefits of the intervention based on likely reductions in the numbers of babies suffering from otitis media, infectious or non-infectious gastroenteritis, NEC and asthma and hence the reduction in the utilisation of health care resources. Unit costs for all except NEC costs were taken from the NICE costing studies [33,34], and uplifted to 2010/11 prices. The gastroenteritis costs are also from the NICE costing study, for which a range is available, a conservative using the lower of the range and a maximum using the upper of the range. Because the analysis was very sensitive to cases of NEC, we used a conservative and a maximum cost range for NEC costs. The conservative unit costs for NEC are calculated using length of stay for International Classification of Diseases (ICD) P77 (the ICD code for NEC) to which are applied weighted neonatal per diem costs from 2010/11 reference costs. The maximum unit costs for NEC are calculated using length of stay from Renfrew et al. [15] to which are applied weighted neonatal per diem costs from 2010/11 reference costs. The published evidence indicates that NEC can be very costly to treat in neonatal babies, and whilst we have undertaken sensitivity analysis using conservative costs for NEC, we believe that they will be an underestimate of the true costs.

As Renfrew et al. point out, studies of breastfeeding in the UK have considered a variety of different durations of breastfeeding [4], and as discussed below, breastfeeding duration in the UK presents a complex picture. The outcomes considered in this paper which had the greatest effect on the economic benefits, NEC and gastroenteritis, are immediately linked to whether the baby receives breastmilk or not while in the neonatal unit. The GIRFS programme did not collect follow up data on the length of time for which babies were breastfed after discharge, so for costs relating to infant outcomes, our calculations are based on breastfeeding in the neonatal unit alone. We did not demand a specific level or duration of breastfeeding, but only considered whether the infant was receiving breastmilk on discharge from the unit. In itself, this will reflect very variable lengths of breastfeeding dependent on the infant’s length of stay.

### Maternal outcomes

The risk of breast cancer is affected by whether mothers breastfeed at all, but also by the duration of breastfeeding. We did not collect information about breastfeeding duration in our study. Breastfeeding duration in the UK is complex, with the 2010 Infant Feeding Survey showing a falling off from an 81% rate of initiation, to 55% breastfeeding at 6 weeks, and 34% at 6 months [35]. Many mothers continue past this point, with the World Health Organisation recommending breastfeeding until 2 years [36]. Duration of breastfeeding is also increasing in the UK [35]. With the small numbers involved in this element of the model, we made the conservative and pragmatic assumption that mothers who breastfed would do so for 6 months. More detail on the calculations and assumptions used may be found in the full published report [28].

## Results

### Cost savings related to infant outcomes – kangaroo skin-to-skin care

Table 1 summarises the cost savings attributable to the increases shown in kangaroo skin-to-skin care during the course of this intervention for the additional 800 babies benefitting ranging from £688,000 under assumptions of minimum savings to more than £2.0 m under assumptions of maximum savings.

**Table 1:** **Summary of economic benefits achieved as a result of an additional 800 babies receiving kangaroo skin-to-skin care**

**Benefits achieved**	**Minimum savings (£)**	**Maximum savings (£)**
Reduction in length of stay in neonatal units	562,461	1,227,187
Reduction in hospitalisation of babies	119,491	766,916
Changes in management of babies during urgent transfers	6,184	15,460
**Total potential savings**	**688,136**	**2,009,563**

Assumptions made:

^1^Reduction in length of stay in neonatal units: minimum savings length of stay 1.1 days, maximum savings 2.4 days;

^2^Reduction in hospitalisation: minimum savings only take costs of severe infections, maximum costs total savings for all conditions;

^3^Changes in management of babies during transfers: minimum savings assume 10% need emergency transport; maximum savings assume 25% need emergency transport.

### Cost savings related to infant outcomes – breastfeeding

Tables 2 and 3 summarise the cost savings to the health services as a result of the increases in breastfeeding during this intervention. The cost savings derive from a reduction in morbidity from a range of conditions, under minimum and maximum cost assumptions. Table 4 summarises the savings across differing assumptions for uptake of breastfeeding.

**Table 2:** **Potential reduction in costs associated with an increase in breastfeeding under conservative cost assumptions following the Getting It Right From the Start intervention**

Clinical condition*	**Unit cost (£)**	**North trent network**		**Yorkshire & Humber**		**Total notional reduction in costs**
		**(47%)**		**Network (52%)**		
		**Reduction in no of babies affected by condition**	**Notional reduction in costs (£)**	**Reduction in no of babies affected by condition**	**Notional reduction in costs (£)**	
**Otitis media**	**37**	**10**	**370**	**175**	**6,488**	**6,858**
**Infectious or non infectious gastroenteritis**	**830**	**2**	**1,660**	**40**	**33,149**	**34,809**
**NEC in LBW infants**	**6,390**	**1**	**6,390**	**13**	**83,070**	**89,460**
**NEC in VLBW infants**	**15,464**	**0**	**0**	**2**	**29,624**	**29,624**
**Asthma**	**37**	**1**	**37**	**16**	**577**	**614**

*NEC: necrotising enterocolitis.

LBW: low birth weight.

VLBW: very low birth weight.

**Table 3:** **Potential reduction in costs associated with an increase in breastfeeding under maximum cost assumptions following the Getting It Right From the Start intervention**

Clinical condition*	**Unit cost (£)**	**North trent network**		**Yorkshire & Humber**		**Total notional reduction in costs**
		**(47%)**		**Network (52%)**		
		**Reduction in no of babies affected by condition**	**Notional reduction in costs (£)**	**Reduction in no of babies affected by condition**	**Notional reduction in costs (£)**	
**Otitis media**	**37**	**10**	**370**	**175**	**6,471**	**6,840**
**Infectious or non infectious gastroenteritis**	**1,599**	**2**	**3,198**	**40**	**63,862**	**67,060**
**NEC in LBW infants**	**29,075**	**1**	**29,075**	**13**	**377,975**	**407,050**
**NEC in VLBW infants**	**48,884**	**0**	**0**	**2**	**93,646**	**93,646**
**Asthma**	**1,753**	**1**	**1,753**	**16**	**27,345**	**29,097**

*NEC: necrotising enterocolitis.

LBW: low birth weight.

VLBW: very low birth weight.

**Table 4:** **Range of estimated cost reductions under different assumptions for costs and clinical conditions and for increases in breastfeeding rates**

**Cost assumptions**	Assumptions on clinical conditions*	**Notional reduction in costs assuming differing breastfeeding rates**		
		**North Trent 47% Yorkshire & Humber 45%**	**North Trent 47% Yorkshire & Humber 50%**	**North Trent 47% Yorkshire & Humber 52%**
Conservative costs	NEC has been taken into account within gastroenteritis calculations	£25,264	£35,340	£42,281
Conservative costs	NEC has been calculated separately and residual allocated to gastroenteritis	£123,742	£132,738	£139,836
Maximum costs	NEC has been taken into account within gastroenteritis calculations	£61,635	£86,260	£102,997
Maximum costs	NEC has been calculated separately and residual allocated to gastroenteritis	£490,714	£530,023	£562,217

*NEC: necrotising enterocolitis.

The largest contribution to cost reductions stems from reductions in gastroenteritis and necrotising enterocolitis (NEC). This is encouraging as these conditions are most directly related to the method of feeding on the neonatal ward, and thus the cost reductions are most tangible and may even result in cash-releasing savings for services. Treatment for NEC, as reflected in the Department of Health’s reference costs, can be extremely resource intensive.

The cost reduction associated with reduction in cases of NEC is £119,084 under minimum cost assumptions and £500,696 under maximum cost assumptions, under our baseline assumptions for increases in breastfeeding rates. The reduction associated with gastroenteritis is £34,809 under minimum cost assumptions and £67,060 under maximum cost assumptions.

### Cost savings related to maternal outcomes – breastfeeding

We calculated that five cases of breast cancer might be avoided by the increases in breastfeeding shown in this study. Published data by Renfrew et al. [4] indicate that the average cost of treating a case of breast cancer (uplifted from their 2009–10 prices) is £12,031. Therefore the estimated notional cost saving from treating the breast cancer cases is £56,154, discounting over 30 years (at a rate of 3.5%) to give a present value of £20,215.

### Overall cost savings – breastfeeding

Table 5 summarises the estimated economic savings associated with increases in breastfeeding as a result of the GIRFS intervention. The greatest contribution was made by a reduction in the hospitalisation of infants, as described above, for which the minimum cost saving was £42,281 and the maximum £562,217. These would also be the most immediate savings. A small contribution was made by cases of breast cancer avoided. Overall the minimum cost saving created by the increase in breastfeeding demonstrated in this study was £62,496 and the maximum was £582,432. Even under our sensitivity analysis with lower breastfeeding rates, the savings would be between £45,479 and £510, 929.

**Table 5:** **Summary of economic benefits achieved as a result of an increase in breastfeeding rates**

**Benefits achieved**	**Minimum savings (£)**	**Maximum savings (£)**
Reduction in hospitalisation of babies	42,281	562,217
Reduction in number of mothers developing breast cancer	20,215	20,215
**Total potential savings**	**62,496**	**582,432**

### Overall costs benefit ratios

The MIHC theme of the regional HIEC programme was funded to a total of £389,000. The theme covered the neonatal work as described in this paper, and two additional workstreams. One of these carried out a similar programme of interventions in maternity care, and one focused on vulnerable women, especially childbearing women in prison.

Taking this into account, the total funding for the programme was notionally divided into four areas, two of which were relevant to the neonatal programme:

- Maternity workstream
- Kangaroo skin-to-skin care
- Breastfeeding in neonatal care
- Vulnerable women and women in prison

Each area was allocated 25% of the costs, meaning that the costs for the neonatal element of the GIRFS programme were calculated at £194,500.

In calculating a cost benefit ratio, we made simple assumptions that the costs and benefits were achieved in the same year (although this would not be the case for any reduction in breast cancer).

Table 6 shows the cost benefit ratio for kangaroo skin-to-skin care under the assumptions of minimum and maximum savings, demonstrating that even under the minimum savings assumption, £7.40 of benefit is achieved for every £1 invested in the kangaroo skin-to-skin care programme. This rises to £21.70 of benefit for the maximum savings assumption.

**Table 6:** **Cost benefit ratio for kangaroo skin-to-skin care**

**Assumptions**	**Economic benefits**	**Programme costs**	**Cost benefit ratio**
Minimum savings	£719,653	£97,250	1:7.4
Maximum savings	£2,106,539	£97,250	1:21.7

Table 7 shows the cost benefit ratio for breastfeeding under the assumptions of minimum and maximum savings. Under the minimum savings assumption, £0.64 of benefit (or savings) is achieved for every £1 invested in the breastfeeding programme. This rises to £6.00 of benefit for the maximum savings assumption. Table 8 shows the calculations assuming the lower increase in breastfeeding rates, giving under the minimum savings assumption, £0.47 of benefit (or savings) achieved for every £1 invested in the breastfeeding programme, rising to £5.25 of benefit for the maximum savings assumption.

**Table 7:** **Cost benefit ratio for breastfeeding, assuming average rate of 50%**

**Assumptions**	**Economic benefits**	**Programme costs**	**Cost benefit ratio**
Minimum savings	£62,496	£97,250	1:0.64
Maximum savings	£582,432	£97,250	1:6.0

**Table 8:** **Cost benefit ratio for breastfeeding, assuming lower rate of 45% for Yorkshire & Humber network**

**Assumptions**	**Economic benefits**	**Programme costs**	**Cost benefit ratio**
Minimum savings	£45,479	£97,250	1:0.47
Maximum savings	£510,929	£97,250	1:5.25

Combining the two aspects, as shown in Table 9, an overall calculation of the costs and benefits of the GIRFS intervention in this area would suggest that for every £1 invested, £4.02 of benefit is generated under minimum savings assumptions and £13.82 of benefit under the maximum savings assumptions. These calculations assume that there would be no double counting across programmes, although this may not be the case for the impact of kangaroo skin-to-skin care and breastfeeding on infant morbidity where there are likely to be shared costs. We can address the problem of potential shared costs in our calculations by assuming that all of the potential resource savings associated with the reduction in hospitalisation of babies has been taken into account in the skin-to-skin care calculations. Table 10 shows the overall cost benefit ratio taking account of shared costs suggesting that £3.93 of benefit is generated under minimum savings assumptions and £13.46 of benefit under the maximum savings assumptions.

**Table 9:** **Overall cost benefit ratio for neonatal element of Getting It Right From the Start programme**

**Assumptions**	**Economic benefits**	**Programme costs**	**Cost benefit ratio**
Minimum savings	£782,419	£194,500	1:4.02
Maximum savings	£2,688,971	£194,500	1:13.82

**Table 10:** **Overall cost benefit ratio for neonatal element of Getting It Right From the Start programme, taking account of shared costs**

**Assumptions**	**Economic benefits**	**Programme costs**	**Cost benefit ratio**
Minimum savings	£708,351	£194,500	1:3.93
Maximum savings	£2,029,778	£194,500	1:13.46

It is important to recognize that our calculations only recognise the resource implications for health services. We have taken no account of the potential reductions in costs faced by families where the mother breastfeeds, for example the cost of formula, of bottles and of sterilizing equipment. Nor have we placed any financial value on the qualitative benefits of skin to skin care or breastfeeding. Therefore all of our costs, including those for treating NEC, are underestimates.

## Discussion

### What is already known on this topic

Significant research studies have already used techniques of economic modelling to demonstrate the potential economic benefits of interventions to increase breastfeeding [1-4]. Rice et al. [37] and Renfrew et al. [15] have demonstrated the economic benefits to be gained from increasing breastfeeding in neonatal care.

### What this study adds

This study complements existing economic models by presenting an economic analysis of pragmatic data from an actual project. It demonstrates that interventions to increase breastfeeding in neonatal units have the potential to generate very significant economic benefits. The particular multifaceted intervention used by the GIRFS programme, described in the published report [9], is shown to have substantial economic benefits compared to costs. GIRFS was comparatively resource-light as described in the Methods section. It used an approach grounded in engagement and participation, with local units taking ownership of the changes. This economic study suggests that such a structure and methodology is likely to provide significant benefits without demanding a large or complex outlay.

The analysis also practically demonstrated the importance of kangaroo skin-to-skin care for preterm babies. While existing evidence has amply described the benefits of kangaroo skin-to-skin care in developing countries, there is less clarity surrounding its benefits in technological healthcare systems, and ultimately its economic returns. This study clearly shows that efforts to increase the proportion of babies receiving kangaroo skin-to-skin care are amply justified by economic as well as clinical and psychological benefits.

### Limitations of this study

The data upon which the economic study was based were collected as part of a pragmatic and uncontrolled intervention whose primary aim was to support units in improving practice. In one sense, our audit of kangaroo skin-to-skin care was groundbreaking, as there have to date been no routinely available data on kangaroo skin-to-skin care. As a result of the GIRFS intervention, a new field has been developed on BadgerNet [32], the clinical record keeping system, to record kangaroo skin-to-skin care. In future UK studies, this may prove to be a useful source of more complete data.

However, there were significant caveats about the accuracy and completeness of the data collected through such an audit. Not all units submitted data every week for example, although in all the weeks on which our calculations were based, at least 85 babies were included in the audit. Most significant is our inability to identify individual babies in the audit, meaning that we cannot adjust for the near-certainty that babies staying more than a week in neonatal care have been counted more than once in consecutive audits. We considered calculating an adjustment factor based on average length of stay, but suggested mean lengths of stay in the published evidence vary considerably. In the event we adjusted for this by reducing our estimate of the numbers of babies benefiting from kangaroo skin-to-skin care by 50%, to arrive at an estimate we considered conservative.

We were also unable to distinguish between different groups of babies eg by gestation, to establish whether the benefits differed for babies with different characteristics. This ability would have enhanced the study’s conclusions.

Similarly our reliance on routine data from BadgerNet to obtain information about breastmilk feeding rates at discharge was complicated by the initial incompleteness of the data set. Complete datasets at baseline and post-evaluation would have made the study’s conclusions more robust.

However, in our economic analysis we have adopted conservative assumptions to take account of the pragmatic nature of the data, and have undertaken a sensitivity analysis to test the impact of different levels of breastfeeding rates. The economic analysis also adopts maximum and minimum assumptions to take account of the range of possible benefits that may accrue, and the real difficulty in estimating these accurately. We are therefore confident that the cost-benefit assumptions we postulate are conservative rather than unrealistic, particularly if the minimum assumptions are considered.

As always in such analyses, the extent to which the economic benefits described translate into the much sought after ‘cash releasing savings’ is debatable. The economic benefits postulated from the increase in kangaroo skin-to-skin care were manifold and will repay dividends in different sectors and at different times, as will those from breastfeeding. The benefits postulated from a reduction in breast cancer, for example, are likely to be generated many years hence, and have thus been significantly discounted.

## Conclusions

This study describes how a real intervention in neonatal units in a region of the UK not only generated significant clinical benefits, but also proved itself to be more than cost-effective even with very conservative assumptions. Further implementation and evaluation of similar interventions, based on the change methodology used, is strongly warranted. There is also a strong case for the inclusion of economic evaluation in future studies of interventions to improve breastfeeding, to build understanding of the short and long term economic benefits of such interventions in a real life context.

## Abbreviations

(ChiMat): National Child and Maternal Health Intelligence Network
(GIRFS): Getting It Right From the Start programme
(HIEC): Health Innovation and Education Cluster
(ICD): International Classification of Diseases
(MIHC): Maternal and Infant Health and Care
(NEC): Necrotising enterocolitis
(NHS): National Health Service
(WTE): Whole time equivalent
(YHEC): York Health Economics Consortium
